# GIS- and Multivariate-Based Approaches for Assessing Potential Environmental Hazards in Some Areas of Southwestern Saudi Arabia

**DOI:** 10.3390/toxics12080569

**Published:** 2024-08-03

**Authors:** Hassan Alzahrani, Abdelbaset S. El-Sorogy, Abdurraouf Okok, Mohamed S. Shokr

**Affiliations:** 1 Geology and Geophysics Department, College of Science, King Saud University, P.O. Box 2455, Riyadh 11451, Saudi Arabia; hsan@ksu.edu.sa (H.A.); asmohamed@ksu.edu.sa (A.S.E.-S.); 2 Earth Sciences and Engineering Department, Missouri University of Science and Technology, McNutt Hall, 1400 N. Bishop Ave, Rolla, MO 65401, USA; aokt2@umsystem.edu; 3 Soil and Water Department, Faculty of Agriculture, Tanta University, Tanta 31527, Egypt

**Keywords:** risk assessment, PCA, cluster analysis, Al-Baha, CF, PLI

## Abstract

Soil contamination is a major issue that endangers the ecology in most countries. Total concentrations of As, Cd, Co, Cr, Cu, Mn, Ni, Pb, VFe, and Zn were determined by analyzing soil samples from 32 surface soil samples in southwest Saudi Arabia, including certain areas of Al-Baha. Kriging techniques were used to create maps of the distribution of metal. To assess the levels of soil contamination in the research area, principal component analysis (PCA), contamination factors (CF), and pollution load index were used. The results show the stable model gave the best fit to the As and Zn semivariograms. The circular model fits the Cd, Co, and Ni semivariograms the best, while the exponential model fits the Cr, V, and Fe semivariograms the best. For Ni and Pb, respectively, spherical and Gaussian models are fitted. The findings demonstrated two clusters containing different soil heavy metal concentrations. According to the data, there were two different pollution levels in the research region: 36.58% of it is strongly contaminated, while 63.41% of it has a moderate level of contamination (with average levels of these metals 5.28 ± 5.83, 0.81 ± 0.19, 18.65 ± 6.22, 45.15 ± 23.25, 60.55 ± 23.74, 972.30 ± 223.50, 33.45 ± 14.11, 10.05 ± 5.13, 84.15 ± 30.72, 97.40 ± 30.05, and 43,245.00 ± 8942.95 mg kg^−1^ for As, Cd, Co, Cr, Cu, Mn, Ni, Pb, V, Fe, and Zn, respectively). The research area’s poor management practices are reflected in the current results, which raised the concentration of harmful elements in the soil’s surface layers. Ultimately, the outcomes of pollution concentration and spatial distribution maps could aid in informing decision-makers when creating suitable heavy metal mitigation strategies.

## 1. Introduction

Soil pollution is a major challenge that worries decision-makers worldwide [1]. There are five million locations worldwide where heavy metal or metalloid pollution of the soil exists at concentrations higher than allowed [2]. Due to their lengthy half-lives in the environment and inability to be chemically or biologically broken down once deposited into the soil, heavy metals have the potential to contaminate the environment and harm ecosystems [3,4] seriously. Providing the right nutrients for plants in the soil is largely dependent on the quality of the soil, which is why sustainable soil management is associated with sustainable agriculture [5]. Since soil is essential for producing high-quality, healthful meals, it should be improved, protected from contamination, and its fertility level raised to meet the world’s growing agricultural needs sustainably [5]. Heavy metal poisoning in soil is a global problem due to the threats to the ecosystem and biological toxicity [6,7,8]. Because of their persistence, ease of accumulation in sediments, and bioaccumulation in food chains, as well as the fact that they degrade the ecological environment and endanger human health, they are regarded as significant environmental pollutants [9,10,11]. In recent decades, there has been an increasing risk to human health and food security due to the negative impacts of pollutants on crop quality [12]. The Al-Baha region is situated in the southwest of Saudi Arabia, between the Tihama Plain and the Hijaz Mountains, with an area of 10,362 km^2^ [13]. Metals are elements found everywhere in the environment, whether through anthropogenic or natural processes [14]. Metals like Pb, Cr, Cd, Hg, Cu, Ni, and Zn are among those on the priority list, according to the Agency of Toxic Substances and Disease Registry [15]. Metalloids such as As have the propensity to create covalent connections with organic groups, which gives rise to their toxicological characteristics [16]. Heavy metal interpolation has been done using geostatistical methods (kriging/cokriging) in the UK [17] and Ireland [18] and empirical Bayesian kriging in California, the USA [19], and China [20]. Geostatistics is a strong technique for representing the spatial variation of soil properties [18]. Through spatial interpolation, kriging is a potent geostatistical technique that may be used to integrate data into raster maps and analyze the spatial distribution of soil properties [17,21,22]. In essence, it uses variograms and associated factors to determine the spatial structure of soil variables (nugget, sill, and range) [17,21]. Both single and combined pollution indices are seen to be useful quantitative methods for determining the concentration of heavy metals in soils and are crucial for forecasting environmental sustainability in the future, especially when it comes to agriculture [23]. Principal component analysis (PCA) has been used to identify several causes of soil pollution, including industrial and agricultural activities and the percentage of heavy metals causing soil contamination [8]. Another advantage of PCA is its capacity to handle massive volumes of data without being constrained by quantity [24,25]. Another aspect of aggregative hierarchical clustering (AHC) is the distances between samples where most similar points are concentrated in a single cluster. The process of continuously merging the two closest clusters is known as AHC, an unsupervised classification method. The current work intends to define contamination levels using PCA, assess the pollution load index (PLI) of the study region, and map the geographical distribution of several selected heavy metals in some areas of southwest Saudi Arabia (Al-Baha). The goals are to look into soil overall concentrations of As, Cd, Co, Cr, Cu, Mn, Ni, Pb, V, Fe, and Zn in certain regions of Saudi Arabia’s southwest. The statistical analysis used in this study enables the evaluation of cause-and-effect relationships and reveals surpassed levels, making it a valuable tool for locating possible sources of contamination. Our study is one of the few that evaluates heavy metal contamination in the research area, as far as we are aware. The findings offer useful details on the extent of soil contamination in the study area, which policymakers can utilize as a reference for both quantitative and qualitative management purposes.

## 2. Materials and Methods

### 2.1. Site Description

**Figure 1:**
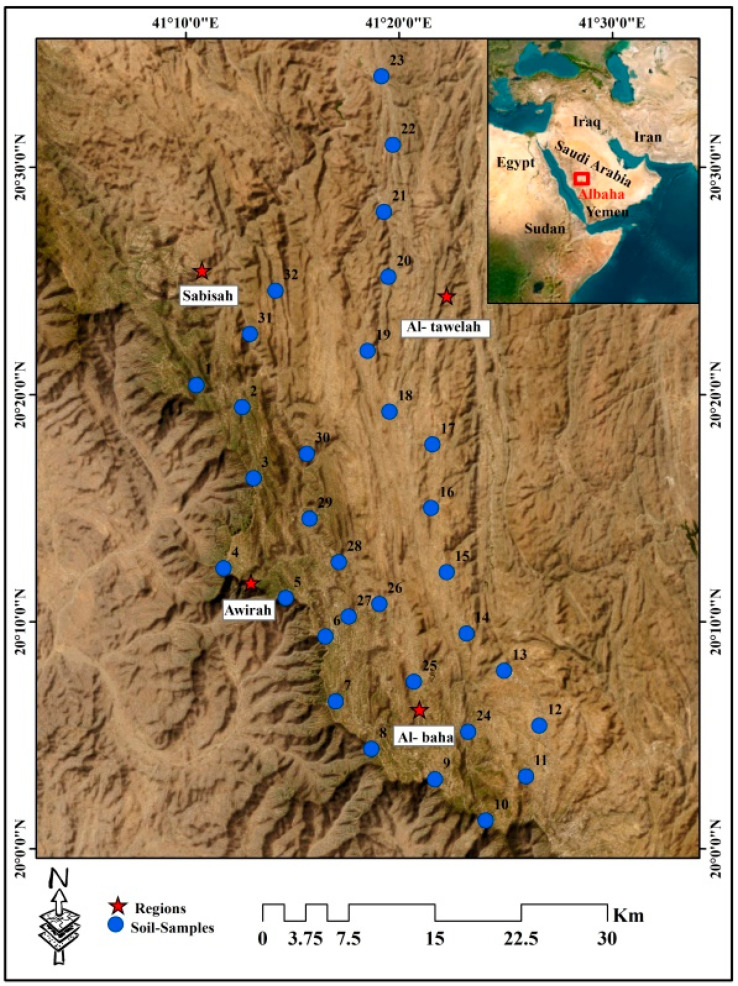
Site description and sample distribution.

The investigated area is located in the northwest of the Al-Baha region between latitudes 19°57′0″ and 20°36′0″ N and longitudes 41°9′0″ and 41°30′0″ E (Figure 1), with an area of 1435.66 km^2^. The Al-Baha region is distinguished by a variety of soil types, plant covers, and topographical and climatic trends. The majority of the crystalline basement of the Al-Baha region, which is situated on the Arabian Shield, is made up of Precambrian continental crust [26]. With an average temperature between 12 and 23 °C, the summer is typically mild to hot, while the winter is warm to chilly. About 150–200 mm of precipitation falls on average each year, and between 50 and 70% of it is moist [27]. The research region is divided geologically into two major Precambrian assemblages: (1) the Jiddah and Ablah groups of metamorphosed coarse clastic and meta-andesitic assemblage, and (2) the Baish and Baha groups of metamorphosed basalt, graywacke, and chert [28], and shanti [29]. The main crops in the study area are wheat, barley, and grape trees (Figure S1).

### 2.2. Collecting Samples and Analytical Procedures

Thirty-two soil samples were collected (Figure 1) using a plastic hand trowel; the samples were taken at a depth of less than 30 cm from the soil’s surface. To provide a representative sample, a composite sample of three subsamples was properly mixed. They were then put in plastic sample bags and kept in an ice box. Inductively coupled plasma-atomic emission spectrometry (ICP–AES) was used in ALS Arabia’s ISO/IEC 17025 (https://www.iso.org/standard/66912.html, accessed on 30 July 2024) (2017)-compliant and accredited to analyze As, Cd, Co, Cr, Cu, Mn, Ni, Pb, V, Fe, and Zn, as per USEPA 3050B [30]. Before chemical analysis, soil samples were sieved and allowed to air dry. A dry, clean Teflon beaker containing around 200 mg of samples was filled with 2 mL of HNO_3_, 6 mL of HCl, and 2 mL of HF after the samples were precisely weighed [31]. The samples underwent a 40-minute digestion process on a heated plate with sand at a mild temperature of 60–120 °C [13]. After filtering, the digest was then put into disposable 25 mL tubes. An empty digest was carried out identically. External calibration was used to do the ICP-AES calibration. Standard stock solutions were utilized, which included a multi-element calibration standard of 1000 mg L^−^^1^. HNO_3_ was used to dilute both the stock and standard solutions. The ICP–AES technique was validated in terms of linearity, limits of quantification (LOQs), and limits of detection (LODs). The correlation coefficient (R^2^) between concentrations and the detector signal for each metal was calculated using linear regression analysis and ranged from 0.95 to 98. The LOQs LODs of each element are illustrated in Table S1.

### 2.3. Contamination Indices

The heavy metal (HM) contamination in soil samples was estimated using the contamination factor (CF) and pollution load index (PLI) [23,32]. According to Hökanson [33], the contamination factor (CF) can be used to express the degree of contamination. The ratio known as the CF is calculated by dividing each metal’s concentration in the sediment by the background or baseline value (Equation (1)). Table S2 shows the CF categorization that is used to assess the levels of soil pollution. Based on element abundances in sedimentary rocks (shale), the background value relates to the baseline concentrations reported by [34]. Contamination factor (CF) values are multiplied by n numbers. The integrated pollution status of the related hazardous groups at the sampling sites was ascertained using the PLI (Equation (2)). The PLI that Tomlinson [35] proposed gives the local population some knowledge about the amount of a component in the environment. The PLI of a single site is equal to the nth root of the n multiplied values of the contamination factor (CF). Table S3 displays the PLI classification used to evaluate soil pollution levels [36].

(1) $$CF=\frac{C_{metal}}{C_{background}}$$

where:

$C_{metal}$ = total measured concentration of heavy metal.
$C_{background}$ is each metal’s background value.

(2) $$PLI=({CF1*CF2*CF3*\ldots \ldots \ldots \ldots .*\;CFn)}^{1/n}$$

where: CF stands for contamination factor and n is the number of specific heavy metals.

### 2.4. Multivariate Analysis

SPSS 28.0 (IBM, Armonk, NY, USA) and Microsoft Excel 2021 (Sacramento, CA, USA) were used for the statistical analysis of the data. One-way ANOVA was utilized for the analysis of variance (ANOVA) [37]. Z-score calculations were performed to normalize the dataset. Managing environmental data, which typically exhibit aberrant distribution, requires this crucial step [22]. To investigate metal correlations in soils, Pearson’s correlation was then run on the produced z-scores. The principal component (PC) approach, along with Bartlett’s test of sphericity, and the Kaiser–Meyer–Olkin (KMO) method, the samples’ appropriateness for PCA was determined. In the event where KMO values were above 0.5, the data were suitable for PCA [38] (Table S4). A PC was only taken into consideration if its eigenvalue was greater than 1.0 [39].

### 2.5. Geostatistical Models

Using ordinary kriging (OK) with circular, spherical, exponential, and Gaussian models, the GIS geostatistical analyzer was utilized to produce raster maps for soil HM. To generate continuous surface layers, the OK is a sophisticated algorithm that forecasts a property’s value at an unsampled point [40]. Using Equation (3), one may estimate the predicted value $Z\left(x_{0}\right)$ based on measured data Z($x_{i}$), weights of measured values ($\lambda_{i}$) at a particular distance, and the number of predicted values (n) within specific neighbor samples.

(3) $$Z\left(x_{0}\right)=\sum_{i=1}^{n}\lambda_{i}\times Z\left(x_{i}\right)$$

The following is the definition of the exponential function:

(4) $$Ɣ\left(h\right)=\left\{\begin{array}{c} 0,h=0\\ C_{0}+C\left(1-e^{-\frac{h}{a}}\right),\;h>0\; \end{array}\right.$$

It was defined that the Gaussian function is:

(5) $$Ɣ\left(h\right)=\left\{\begin{array}{c} C_{0}+C\left(1-\exp\left(-\frac{h^{2}}{a^{2}}\right)\right),\;h>0\\ 0,h=0 \end{array}\right.$$

The spherical function was defined as:

(6) $$Ɣ\left(h\right)=\left\{\begin{array}{c} C_{0}+C\left(\frac{3h}{2a}-\frac{1}{2}-{\left(\frac{h}{a}\right)}^{3}\right),\;0<h\leq a\\ C_{0}+C,\;h>a\\ 0,\;h=0 \end{array}\right.$$

The definition of the circular function was given as:

(7) $$Ɣ\left(h\right)=\left\{\begin{array}{c} C_{0}+C(1-\frac{2}{\pi }\cos^{-1}\frac{h}{2}+\sqrt{1-(h^{2}/a^{2}})),\;0<h\leq a\\ C_{0}+C,\;h>a\\ 0,\;h=0 \end{array}\right.$$

The real ranges for the spherical, circular, exponential, and Gaussian functions, respectively, are represented by the letters and in these formulas. The nugget is denoted by C_0_, the partial sill by C, and the spatial lag by h. For these variograms, the soil sample spatial variation was isotropic. OK can provide fair approximations with the least amount of error [41]. The nugget (C_0_), partial sill (C), and sill (C_0_ + C) are the spatial variation parameters that are obtained by fitting the experimental semivariograms using multiple models. The nugget, which measures short-range variability, is the semivariogram at a lag distance of zero. The sill represents the overall sample variability and is the point at which the model flattens out [42].

### 2.6. Validation of Geostatistical Analysis

Heavy metal concentrations were mapped using the geostatistical models previously discussed. The mean standardized error (MSE) and the root mean square standardized error (RMSSE) were the prediction errors that were taken into account when using the cross-validation technique to test the validity and efficiency of OK models. The best-fit model was chosen based on the lowest MSE (close to zero) and RMSSE close to unity [43,44]. The mathematical expressions of these errors are as follows [45]. The models were assessed using the following two equations (Equations (8) and (9)).

(8) $$\mathrm{Mean}\;\mathrm{standardized}\;\mathrm{error}\;(\mathrm{MSE})=\frac{1}{N}\sum_{i=1}^{N}\left[Z_{1}\left(X_{1}\right)-Z_{2}\left(X_{2}\right)\right]$$

(9) $$\mathrm{Root}\;\mathrm{mean}\;\mathrm{square}\;\mathrm{standardized}\;\mathrm{error}\;(\mathrm{RMSSE})=\sqrt{\frac{1}{N}\sum_{I=1}^{N}{\left[Z_{1}\left(x_{i}\right)-Z_{2}\left(x_{i}\right)\right]}^{2}}$$

where: $Z_{1}$ ($x_{i}$) is measured values and Z_2_ ($x_{i}$) = predicted values.

## 3. Results and Discussion

### 3.1. Variation of Heavy Metals within the Study Area

Table 1 summarizes the concentrations of heavy metals in the topsoil along the study area. The As has been determined to be between 0.1 and 22 mg kg^−1^. The mean As concentration (7.1 ± 6.3 mg kg^−1^) surpasses the upper crust concentrations by Wedepohl [34], and the recommended levels of heavy metals by DEA [46] (Table 1). The element arsenic is more common in clayey soil types and is generally believed to have a geological origin—earth. However, since man-made sources release arsenic into the environment more frequently than natural ones, there is a considerable quantity of pollution from anthropogenic sources in the environment [47]. The average value of the Cd concentration was 0.8 ± 0.2 mg kg^−1^, with a range of 0.1 to 1.1 mg kg^−1^. Cadmium (Cd) is a trace element that is commonly present in the environment but is not necessary. Anthropogenic and geogenic sources can increase the levels of Cd in soils and groundwater, which is crucial for preserving a wholesome food supply and clean drinking water. High Cd levels can cause cancer in humans [48]. The cobalt concentration ranged from 9 to 35 mg kg^−^^1^, with an average of 20.6 ± 6.6 mg kg^−1^. When cobalt is present at low concentrations, it is essential to the growth of leguminous plants. Due to its inclusion in Vitamin B12, it also offers numerous advantages for human health. However, when used excessively, it can have detrimental effects, such as restricting the nitrogen metabolism and photosynthesis of plants, and it can also have detrimental effects on the human heart and lungs [49]. The average concentration of chromium was 58.2 ± 34.4 mg kg^−1^, ranging from 13 to 1914 mg kg^−1^. One of the harmful heavy metals present in soil naturally is chromium, which is produced by the earth’s crust’s weathering of minerals or by industrial leftovers that seep into the soil. Because it is crucial for the growth of leguminous plants when it is present in low concentrations, cobalt is a crucial component of plant nutrition. Plant limitations in photosynthesis and nitrogen metabolism are just two of the several issues that arise from an increase in cobalt concentration in the soil [50]. One of the primary sources of cobalt contamination is fertilization [51]. The copper mg kg^−^^1^ average is 61.1 ± 20.5. The study area’s soil samples exhibit greater amounts of Cu than both the typical upper earth crust values of Wedepohl [34] and the DEA [46] (Table 1). The primary cause of copper buildup in soils is human activity, such as mining and industrial processes. In agriculture, copper-containing chemicals are commonly utilized, especially in pesticides applied to vineyards and orchards [52]. The concentration of Mn in total varied between 684 and 1565.0 mg kg^−1^, with an average of 1015.2 ± 212 mg kg^−1^. The human body, plants, and animals all rely heavily on manganese (Mn), which is an important cofactor of many enzymes. As an alloying element, it is also an essential raw ingredient. The environment is overexposed to it when it is used extensively for industrial purposes, endangering both public health and the ecosystem [53]. The overall concentration of Ni ranged from 684 to 1565 mg kg^−1^, with a mean of 1015.2 ± 212 mg kg^−1^. The proper development of plants, animals, and soil/water bacteria are just a few of the crucial biological processes that are known to depend on nickel. However, too much nickel can poison wildlife. Researchers have discovered that nickel has an impact on higher plants’ ability to photosynthesize, significantly reducing soil fertility and contributing to a number of chronic human ailments [54]. The range of Pb content was 4–27 mg kg^−1^, with an average of 10.1 ± 5.5 mg kg^−1^. Lead pollution in the soil may be caused by pesticides and fertilizers. Lead deposition in agricultural fields can also result from the usage of lead ammunition for hunting [55]. Vanadium (V) is contained in the entire composition at an average of 89.8 ± 33 mg kg^−1^. According to [56,57], vanadium is a strategically significant metal that is widely used in modern civilization in the manufacturing of steel alloys and sulfuric acid. Vanadium can be found in nature in two different oxidation states: tetravalent and pentavalent [58,59]. Because pentavalent vanadium negatively affects phosphate metabolism, it is more harmful to humans, animals, and plants than tetravalent vanadium [60]. Typical soil iron concentrations range from 0.2% to 55% (20,000 to 550,000 mg/kg^−1^) [61]. Because of different soil types and the existence of additional sources, concentrations can vary greatly, even within localized areas. With an average of 103.08 ± 17.63 mg kg^−1^, the Zn values in the research region varied greatly, from 13 to 77 mg kg^−^^1^. Both humans and plants need zinc, but too much of it can be dangerous [62]. It might inflict immediate damage, which could result in problems with the immune and digestive systems. Copper insufficiency symptoms can also be caused by excessive zinc levels that prevent copper absorption [63]. 

### 3.2. Heavy Metals Distribution within the Investigated Area

**Figure 2:**
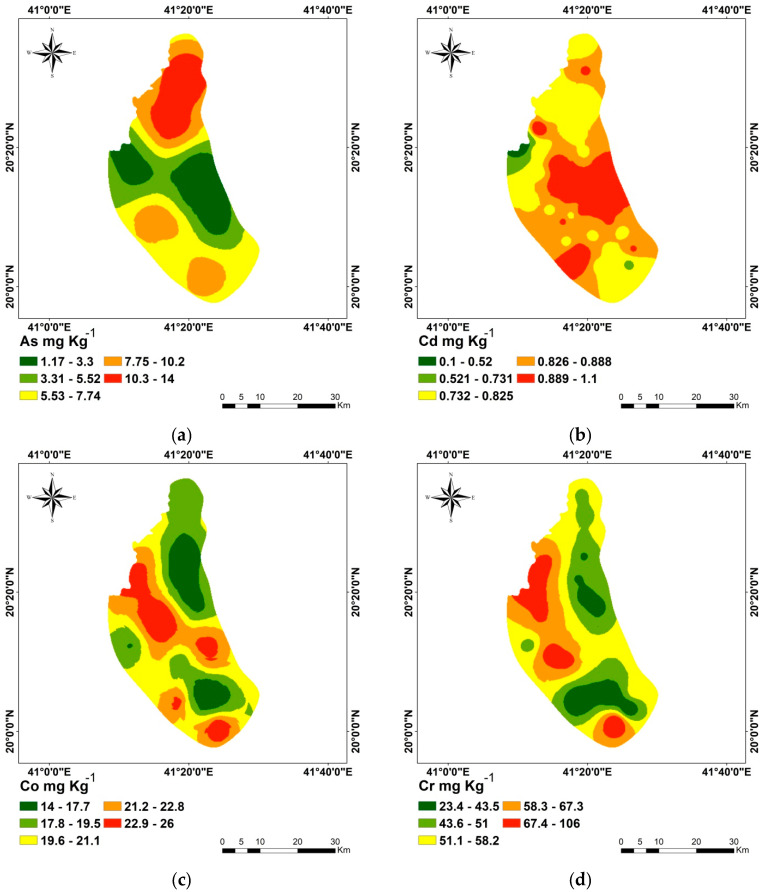

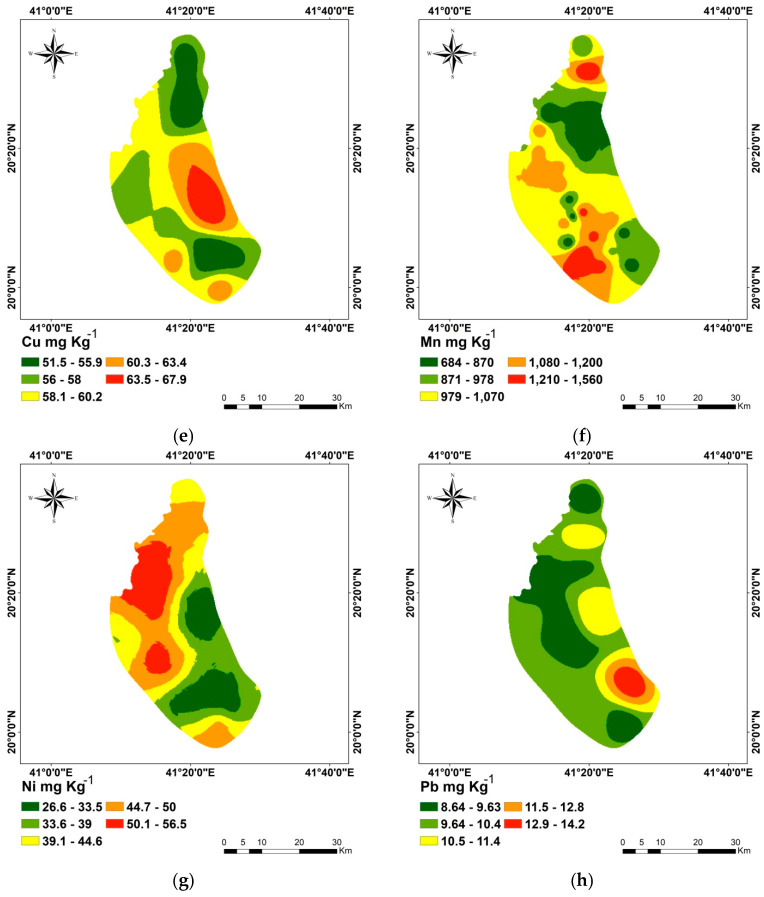

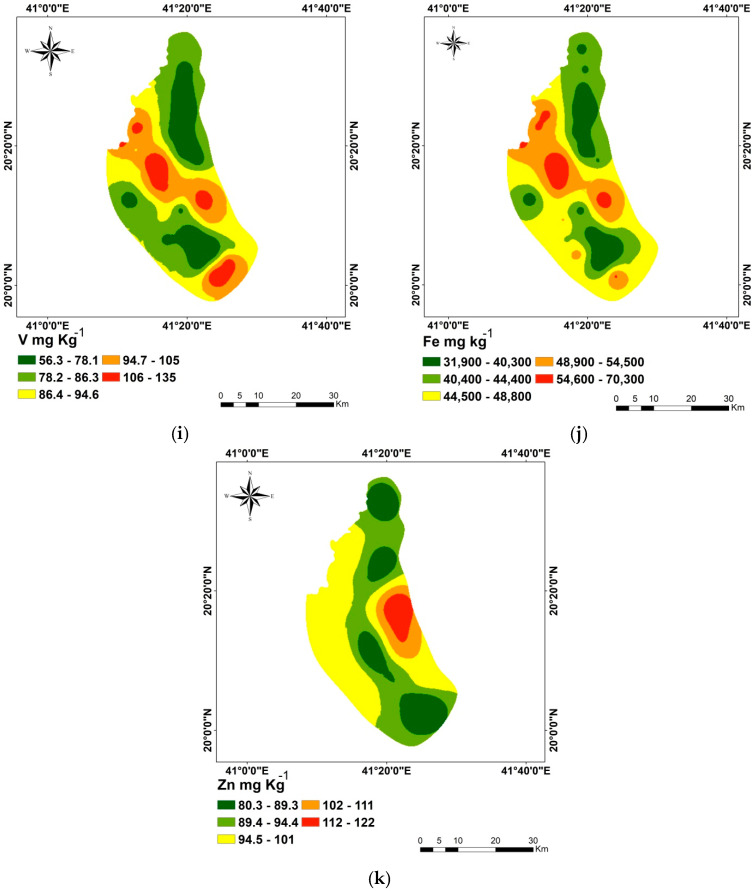
Kriging interpolation maps of studied heavy metals. As (**a**), Cd (**b**), Co (**c**), Cr (**d**), Cu (**e**), Mn (**f**), Ni (**g**), Pb (**h**), V (**i**), Fe (**j**) and Zn (**k**).

Table 2 provides the parameters for the semivariograms (Figure S2) that illustrate the regional distribution of the eleven metals. The stable model was the best model for the As and Zn semivariograms, with the lowest errors. The best model to fit the semivariograms of Cd, Co, and Ni was the circular model; the best model to fit the semivariograms of Cr, V, and Fe was the exponential model. Spherical and Gaussian models are fitted to Ni and Pb, respectively. There are strong correlations between the eleven metals’ measured and anticipated concentrations, according to the semivariogram models’ cross-validation (Figure S2). The prediction errors (Table 2) show that while the RMSSE values were near unity, the MSE values for each applied model were nearly zero. Table 2 illustrates that for every used OK model, an appositive nugget effect (larger than 0) and a sill value were attained. The spatial dependency (SPD) is defined by the nugget/sill ratio; values less than 0.25, 0.25–0.75, and greater than 0.75 indicate, correspondingly, a strong, moderate, and weak SPD [45]. As a result, Fe has a strong SPD, whereas the other elements have a moderate SPD. Similar spatial patterns were seen for the concentrations of Cd, Cr, and Ni, which were delineated by a northwest zone with high values. The eastern parts of the study area showed the highest levels of Cu, Cd, and Zn, while the southern part showed the highest concentrations of Mn and V, and the southern part showed the highest concentrations of Pb. The northern and middle regions of the study area showed the highest concentrations of As and Fe, respectively, Figure 2.

### 3.3. Correlation between Selected Heavy Metals

A linear relationship can be estimated using Pearson’s correlation coefficient. In all of the natural sciences, it is one of the most often utilized statistical values [64]. There were substantial correlations (*p* < 0.05) and highly significant correlations (*p* < 0.01) between different metals (Table 3). The concentration of As is positively correlated with Ni (r = 0.483 **) and negatively correlated with Cu (r = −0.425 *), V (r = −0.367 *), and Fe and Zn (r = −0.360 *). Cd did not significantly correlate with the other metals. Co was correlated with Cu (r= 0.642 **), V (r = 0.824 **), and Ni (r = 0.399 *). The concentration of Mn is positively correlated with Co (r = 0.380) due to their comparable chemical characteristics, cobalt (Co) and manganese (Mn) are closely related in soils [65]. There is a substantial positive association between the chemically related elements Co and Fe (0.920) in the same line of [66]. Cr is correlated positively with Co (r = 0.576 **), V (r = 0.469 **), Cu (r = 0.474 **), Ni (r = 0.716 **), and Fe (r = 0.466 **) [67]. Pb did not significantly correlate with the other metals.

### 3.4. Analysis of Factors Extracted from PCA

**Figure 3:**
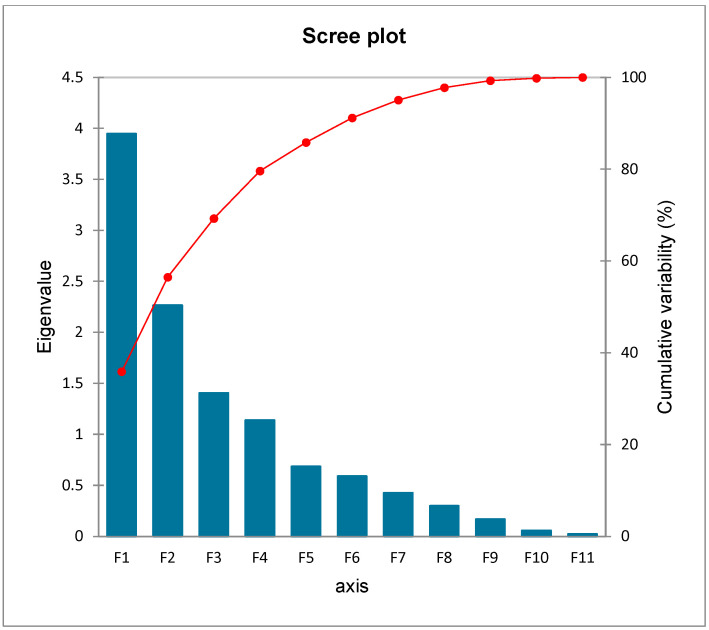
Scree plot of different principal components.

**Figure 4:**
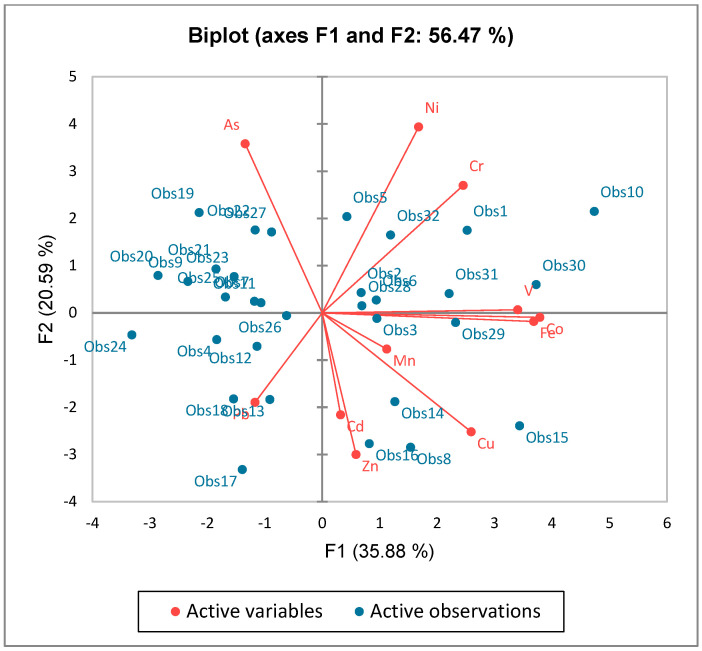
Biplot of principal components.

Table 4 and Table 5 display the PCA results for the metal content in the soil samples. The first eigenvalue results in Table 4 and Figure 3 indicate that factor analysis made it possible to extract four components (F1, F2, F3, and F4). CO (0.972), V (0.874), and Fe (0.945) were shown to be substantially linked with the first component (F1) in the component matrix for the data. Additionally, soil Cr and Cu (0.630 and 0.665), with moderate absolute concentrations, were linked to F1. With moderate values, As (−0.697), Ni (−0.766), and Zn (0.584) were clearly visible in the second component (F2). The levels of Cd (0.657) and Mn (0.703) had a moderate relationship with the third component (F3). (Table 5). There was a moderate correlation between Pb (0.591) and the fourth PC component (F4). F1, which accounted for 35.77% of the total variance, was dominated by CO, Cr, Cu, V, and Fe, indicating that these metals came from comparable sources (Table 4 and Figure 4) [68]. F2 mostly ascribed the variance to As, Ni, and Zn and explained 20.59% of the overall variation. As and Ni had a moderately negative association with F2 while Zn had a moderately positive association with F2, suggesting that their sources might have been distinct [68]. Cd and Mn dominated F3, which accounted for 12.77 percent of the variance in total. Pb dominated F4 and explained 10.35% of the overall variation. The principal component analysis biplot of F1, F2, F3, and F4 is displayed in Figure 4. It rescales the loadings plot and the score plot so that they overlap on a single plot. The biplot displays the variables as arrows, and the correlation between each pair of variables is determined by taking the cosine of the angle produced by the arrows. The narrower the angle between each pair of arrows, the higher the correlation between the variables [69].

### 3.5. Cluster Analysis of Study Area

**Figure 5:**
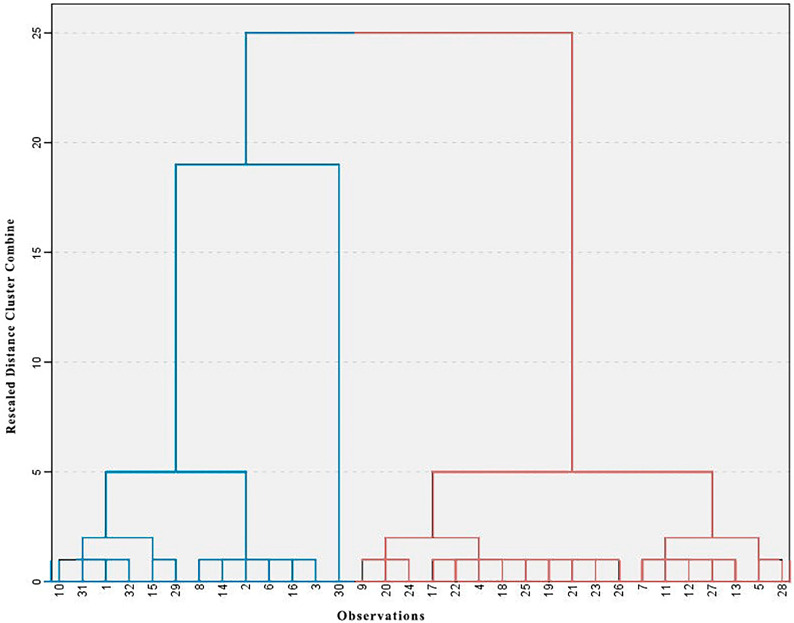
Hierarchical cluster analysis (HCA) extracted from PCA; blue expresses C1, and red represents C2.

A dendrogram representing the findings of the hierarchical cluster analysis (HCA) for the variables is displayed in Figure 5. The first cluster has 13 observations, whereas the second has 19, according to descriptive statistics presented in Table 6. The ranges, means, and standard deviations (SD) of each variable differ. Figure 5 displays the locations of every cluster observation. Using PCA, those two clusters were selected out of factors (F1, F2, F3 and F4). Cluster 1 exhibits greater amounts of Co, Cr, Cu, Mn, V, Fe, and Zn, while cluster 2 displays lower quantities of As and Cd (Table 6).

### 3.6. Hazard Assessment

#### 3.6.1. Contamination Factor (CF)

**Figure 6:**
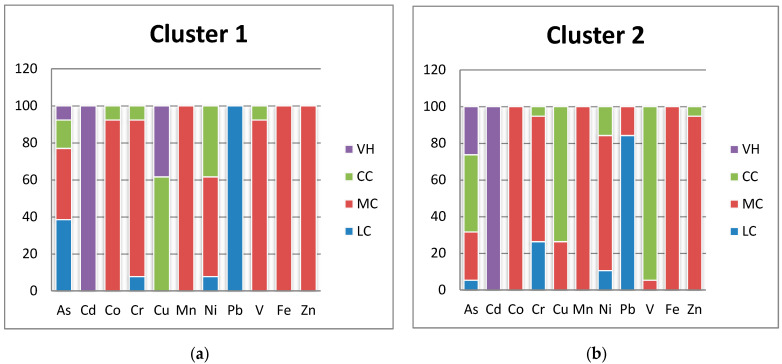
CF for each studied heavy metal in cluster 1 (**a**) and cluster 2 (**b**) (low contamination(LC), moderate contamination (MC), considerable contamination(CC), and VH (very high contamination).

For every cluster, the contamination factors (CFs) were computed to evaluate the likely ecological risk associated with a particular heavy metal. The cluster classification of each site is displayed in Figure 6. According to the results, cluster 1’s contamination factor of As indicated low contamination (38.46%), moderate contamination (38.46%), considerable contamination (15.38%), and very high contamination (7.69%); in contrast, cluster 2’s contamination level was relatively high, with 5.26% of soil samples having low contamination, 26.31% having moderate contamination, 42.10% having considerable contamination, and 26.1% having very high contamination [33], 1980) (Figure 6). In terms of Cd, CF was very high in both clusters. It is possible that human activity and fertilizer application in the research region are to blame for the rise in Cd levels in both clusters [13]. Co pollution was found in cluster 1 at moderate and considerable levels, whereas low, moderate, and considerable contamination levels were found in cluster 2. In comparison, cluster 1 recorded 7.69% low, 84.61% moderate, and 7.69% considerable contamination, whereas, the contamination levels in cluster 2 were 26.31, 68.42, and 5.26%, respectively. A decline in soil quality could have an impact on human health if the amount of chromium in the soil increases [70]. Cluster 1 has a considerable and very high level of contamination, measuring 61.53% and 38.47%, respectively, according to the Cu contamination factor. Additionally, cluster 2 had a relative percentage of 26.31 and 73.38%, respectively, indicating moderate and considerable Cu contamination. Mn pollution was found at moderate levels in both clusters. Cluster 1 has a moderate level of contamination, as indicated by the Ni contamination factor of 53.84%. In addition, cluster 2 had a relative proportion of 73.68. According to the findings, Pb contamination in cluster 1 was found to be low (100%). Cluster 2 showed low (84.21%) to moderate levels of contamination (15.78%). While cluster 1 recorded 92.3% moderate contamination and 7.69% considerable contamination by V, cluster 2 recorded 5.26% moderate contamination and 94.73% considerable contamination. Zn and Fe had moderate levels of contamination in cluster 1, whereas moderate, moderate, and considerable amounts of contamination were found in cluster 2, respectively (Figure 6).

#### 3.6.2. Pollution Load Index (PLI)

**Figure 7:**
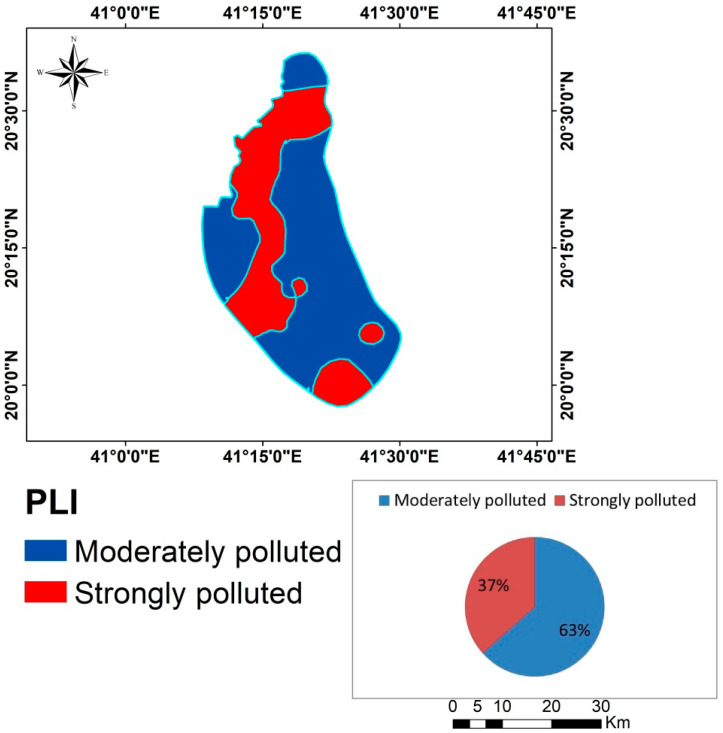
Map of PLI in the study area.

There were two levels of pollution in the research region, as shown in Table 7 and Figure 7. Some 36.58% of the research area is significantly contaminated, whereas 63.41% of the region indicated a moderate level of pollution. Moderate concentrations of heavy metals were found in the majority of the study area, with average levels of these metals 5.28 ± 5.83, 0.81 ± 0.19, 18.65 ± 6.22, 45.15 ± 23.25, 60.55 ± 23.74, 972.30 ± 223.50, 33.45 ± 14.11, 10.05 ± 5.13, 84.15 ± 30.72, 97.40 ± 30.05, and 43,245.00 ± 8942.95 mg kg^−1^ for As, Cd, Co, Cr, Cu, Mn, Ni, Pb, V, Fe, and Zn, respectively. Anthropogenic sources for Cu, Zn, Cd, and Pb may have originated from sewage and agricultural practices; natural sources for As, Cr, Mn, Fe, Co, and Ni, mainly from the weathering of soil minerals and atmospheric deposition [13]. Farmers should use manure and biofertilizers, which have different levels of heavy metals and patterns, to reduce their dependency on chemical pesticides and fertilizers [13]. Perhaps they could use beneficial microbes that could drastically lower the levels of soil contamination [71].

## 4. Conclusions

The current study focuses on the heavy metal contamination assessment of the soil in the southwest region of Saudi Arabia, which includes some parts of Al-Baha. This pollution is seen to be one of the major barriers to food security and sustainable development. This study proved that semivariogram models are a useful tool for forecasting the heavy metals’ spatial distribution maps in the investigated area. In addition, the combination of PCA and HCA produced unexpected outcomes by creating two zones within the study area, each with a different heavy metal content and pattern. The findings revealed two degrees of heavy metal pollution: moderate and strong. A moderate amount of pollution was found in roughly 63.41% of the research region. The majority of the pollution levels were above the threshold of their average concentration in the parent material and the crust of the planet. In order to decrease their reliance on chemical pesticides and fertilizers, farmers ought to employ manure and biofertilizers that differ in terms of heavy metal concentration and pattern. To prevent and regulate rising levels of heavy metals (HMs), especially those of Cu, Zn, Cd, and Pb, soil concentrations in the research region should be routinely monitored. It also shows that in areas with limited data availability, a combination of statistical and geostatistical approaches works well and is simple to apply. To improve the geographic interpretation of soil contamination, it is advised to increase the sampling size. The study concludes by urging the implementation of farm management legislation to curtail harmful human behaviors that worsen environmental contamination. Additionally, research in the future will concentrate on managing and lessening the consequences of soil pollution.

## Figures and Tables

**Table 1:** Descriptive statistics of the studied heavy metals.

								Recommended Concentrations (mg kg^−1^)	
Elements	Concentrations	N	Min.	Max.	Mean	Skewness	Kurtosis	Upper Crust Concentrations [34]	Recommended Concentrations [46]
As	mg kg^−1^	32	0.1	22.0	7.1 ± 6.3	0.8	−0.1	2	5.8
Cd			0.1	1.1	0.8 ± 0.2	−3.3	16.3	0.1	7.5
Co			9	35.0	20.6 ± 6.6	0.4	−0.6	11.6	300
Cr			13	191.0	58.2 ± 34.4	2.0	6.1	35	6.5
Cu			30	110.0	61.1 ± 20.5	0.9	0.0	14.3	16
Mn			684	1565.0	1015.2 ± 212	0.6	0.0	527	740
Ni			12	72.0	42.3 ± 17.2	0.0	−0.9	62	91
Pb			4	27.0	10.1 ± 5.5	1.8	3.5	17	20
V			39	163.0	89.8 ± 33	0.7	−0.5	53	150
Fe			31,700	70,700	45,846.9 ± 10,041.7	0.6	−0.5	59,100	n/a
Zn			60	191.0	96.7 ± 24.7	1.9	6.2	52	240

**Table 2:** The spatial data modelling semivariogram.

Elements	Concentrations	Model	Nugget	Partial Sill	Sill	Nugget/ Sill	SPD	MSE	RMSSE
As	mg kg^−1^	Stable	0.5	0.4	0.90	0.55	Moderate	−0.008	0.98
Cd		Circular	0.51	0.41	0.92	0.55	Moderate	0.034	1.22
Co		Circular	0.54	0.46	1	0.54	Moderate	0.005	0.99
Cr		Exponential	0.41	0.64	1.05	0.39	Moderate	0.011	1.07
Cu		Spherical	0.7	0.27	0.97	0.72	Moderate	−0.008	1.008
Mn		Gaussian	0.50	0.45	0.95	0.52	Moderate	0.006	1.004
Ni		Circular	0.51	0.48	0.99	0.51	Moderate	0.005	1.01
Pb		Gaussian	0.23	0.53	0.76	0.30	Moderate	−0.003	0.99
V		Exponential	0.38	0.63	1.01	0.37	Moderate	0.017	0.98
Fe		Exponential	0.01	0.95	0.96	0.01	Strong	0.019	0.91
Zn		Stable	0.55	0.29	0.84	0.65	Moderate	−0.03	1.05

**Table 3:** Pearson correlation between different studied metals.

Variables	As	Cd	Co	Cr	Cu	Mn	Ni	Pb	V	Zn	Fe
As	1	−0.007	−0.309	0.099	−0.425 *	−0.014	0.483 **	−0.060	−0.367 *	−0.360 *	−0.360 *
Cd	−0.007	1	0.134	−0.254	0.258	0.320	−0.205	0.047	0.018	0.068	0.086
Co	−0.309	0.134	1	0.576 **	0.642 **	0.380 *	0.399 *	−0.240	0.824 **	0.095	0.920 **
Cr	0.099	−0.254	0.576 **	1	0.235	0.004	0.716 **	−0.137	0.469 **	−0.048	0.466 **
Cu	−0.425 *	0.258	0.642 **	0.235	1	0.238	−0.050	0.033	0.474 **	0.391 *	0.539 **
Mn	−0.014	0.320	0.380 *	0.004	0.238	1	0.029	−0.130	0.046	−0.024	0.211
Ni	0.483 **	−0.205	0.399 *	0.716 **	−0.050	0.029	1	−0.209	0.251	−0.156	0.402 *
Pb	−0.060	0.047	−0.240	−0.137	0.033	−0.130	−0.209	1	−0.311	0.320	−0.277
V	−0.367 *	0.018	0.824 **	0.469 **	0.474 **	0.046	0.251	−0.311	1	−0.018	0.871 **
Zn	−0.360 *	0.068	0.095	−0.048	0.391 *	−0.024	−0.156	0.320	−0.018	1	0.189
Fe	−0.360 *	0.086	0.920 **	0.466 **	0.539 **	0.211	0.402 *	**−0.277**	0.871 **	0.189	1

Note: * *p* < 0.05; ** *p* < 0.01. Bold = significance.

**Table 4:** Eigenvalue of factors extracted by PCA.

	Factor Loading										
	F1	F2	F3	F4	F5	F6	F7	F8	F9	F10	F11
Eigenvalue	**3.947**	**2.265**	**1.405**	**1.139**	0.685	0.589	0.425	0.299	0.168	0.057	0.022
Variability (%)	**35.877**	**20.594**	**12.771**	**10.358**	6.223	5.357	3.863	2.715	1.524	0.518	0.199
Cumulative %	**35.877**	**56.472**	**69.243**	**79.601**	85.824	91.180	95.044	97.759	99.282	99.801	100.000

Bold = significance.

**Table 5:** Factors extracted by PCA for studied heavy metals.

Elements	Concentrations	Component			
		1	2	3	4
As	mg kg ^−1^	−0.344	**−0.697**	0.276	0.402
Cd		0.082	0.420	**0.657**	0.256
Co		**0.972**	0.018	0.095	0.045
Cr		**0.630**	−0.525	−0.274	0.256
Cu		**0.665**	0.490	−0.014	0.187
Mn		0.288	0.149	**0.703**	0.250
Ni		0.431	**−0.766**	−0.065	0.367
Pb		−0.300	0.369	−0.370	**0.591**
V		**0.874**	−0.013	−0.077	−0.302
Zn		0.151	**0.584**	−0.412	0.409
Fe		**0.945**	0.035	−0.027	−0.066

Note: Bold values match for each variable to the factor for which the squared cosine is the largest.

**Table 6:** Quantitative data of the studied elements for two clusters (C1 and C2).

Clusters	Statistics	As	Cd	Co	Cr	Cu	Mn	Ni	Pb	V	Fe	Zn
		mg kg^−1^										
C1	n	13										
	Min	0.1	0.1	22	13	52	769	14	5	85	49,900	77
	Max	15	1.1	35	191	110	1565	70	16	163	70,700	144
	Mean	4.19 ± 4.54 ^a^	0.86 ± 0.25 ^a^	27.08 ± 4.21 ^a^	75.08 ± 42.51 ^a^	74.77 ± 20.21 ^a^	1109.31 ± 178.73 ^a^	48.31 ± 18.08 ^a^	8.62 ± 3.50 ^a^	116.23 ± 27.2 ^a^	56,276 ± 6075.24 ^a^	103.08 ± 17.63 ^a^
	Skewness	9.16 ± 6.57 ^b^	0.82 ± 0.05 ^a^	16.16 ± 3.51 ^b^	46.58 ± 21.91 ^b^	51.74 ± 14.93 ^b^	950.84 ± 212.88 ^b^	38.11 ± 15.80 ^a^	11.11 ± 6.36 ^a^	71.79 ± 22.92 ^b^	38,710.53 ± 4090.35 ^b^	92.32 ± 28.11 ^a^
	Kurtosis	1.138	−2.616	0.387	1.588	0.602	0.861	−0.547	0.986	0.384	1.098	0.936
C2	n	19										
	Min	0.1	0.7	9	17	30	684	12	4	39	31,700	60
	Max	22	0.9	24	107	91	1425	72	27	139	47,300	191
	Mean	9.16 ± 6.57 ^b^	0.82 ± 0.05 ^a^	16.16 ± 3.51 ^b^	46.58 ± 21.91 ^b^	51.74 ± 14.93 ^b^	950.84 ± 212.88 ^b^	38.11 ± 15.80 ^a^	11.11 ± 6.36 ^a^	71.79 ± 22.92 ^b^	38,710.53 ± 4090.35 ^b^	92.32 ± 28.11 ^a^
	Skewness	0.57	0.22	0.24	1.34	1.04	1.01	0.28	1.57	1.7	0.38	2.46
	Kurtosis	−0.72	0.31	0.79	2.33	1.24	0.01	−0.18	2.09	3.42	−0.27	8.54

Note: There is a considerable discrepancy between the means of variables with different letters.

**Table 7:** The mean concentration of heavy metals in every level of pollution in the research area.

Pollution Level	As	Cd	Co	Cr	Cu	Mn	Ni	Pb	V	Fe	Zn	Area km^2^, %
mg kg^−1^												
M	5.28 ± 5.83	0.81 ± 0.19	18.65 ± 6.22	45.15 ± 23.25	60.55 ± 23.74	972.30 ± 223.50	33.45 ± 14.11	10.05 ± 5.13	84.15 ± 30.72	43,245.00 ± 8942.95	97.40 ± 30.05	910.41, (63.41%)
S	10.25 ± 5.92	0.883 ± 0.08	23.83 ± 6.17	79.83 ± 39.67	62.00 ± 14.42	1086.75 ± 177.23	56.92 ± 10.90	10.17 ± 6.20	99.33 ± 35.68	50,183.33 ± 10,639.96	95.50 ± 12.34	525.19, (36.58%)

## Data Availability

All data are included in the manuscript and supplementary files.

## Supplementary Materials

The following supporting information can be downloaded at https://www.mdpi.com/article/10.3390/toxics12080569/s1, Figure S1: Some cultivated crops in the study area; Figure S2: Semivariograms of studied elements; Table S1: Limit of detection of studied elements; Table S2: Classification of contamination factor(CF); Table S3: Classification of pollution load index (PLI); Table S4: KMO and Bartlett’s Test for studied variables.
