# Supplementary material for: GIS- and Multivariate-Based Approaches for Assessing Potential Environmental Hazards in Some Areas of Southwestern Saudi Arabia
Source: Toxics. 2024 Aug 3;12(8):569. doi: 10.3390/toxics12080569 (PMC11359128; doi:10.3390/toxics12080569)
Supplement: Supplementary file 1 [file toxics-12-00569-s001.zip › toxics-3091073-supplementary.pdf]

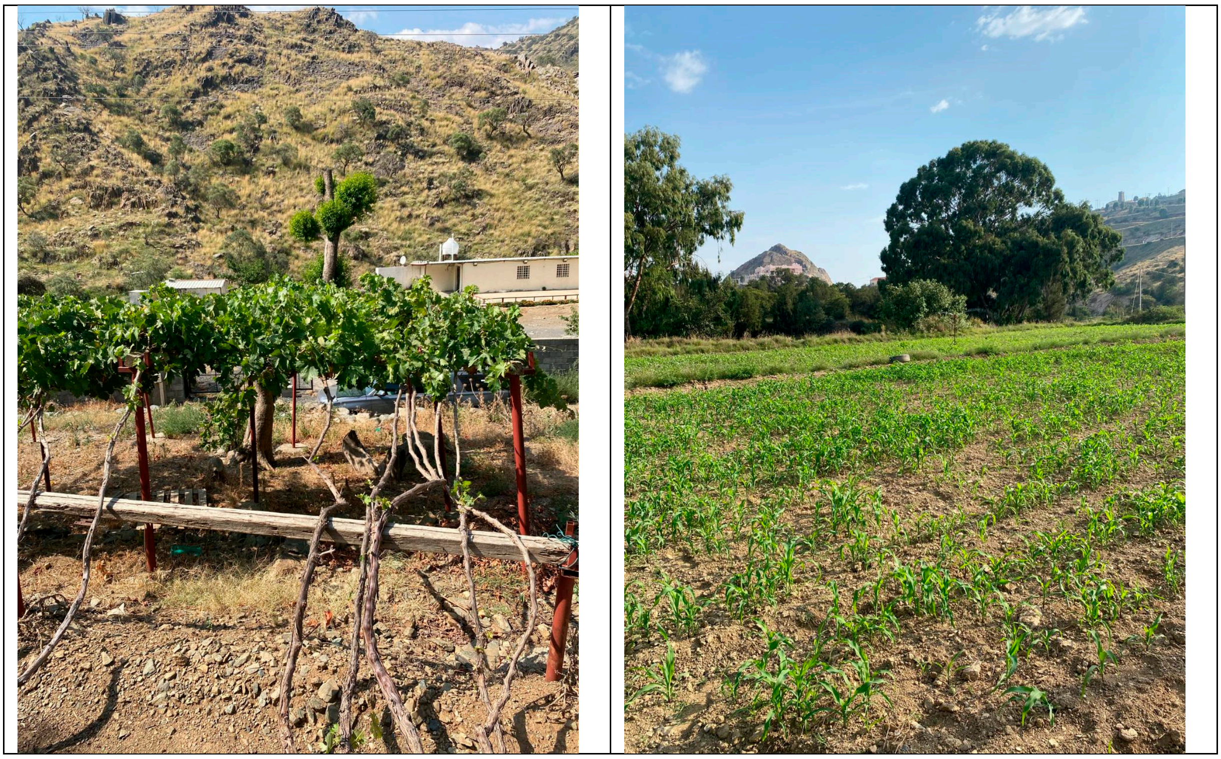

Figure S1. Some cultivated crops in the study area.

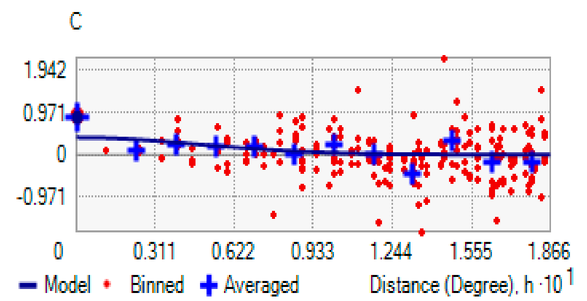

As

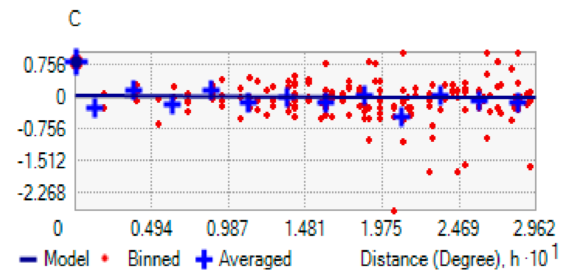

Cd

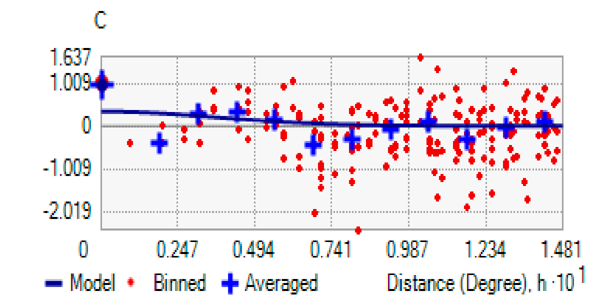

Co

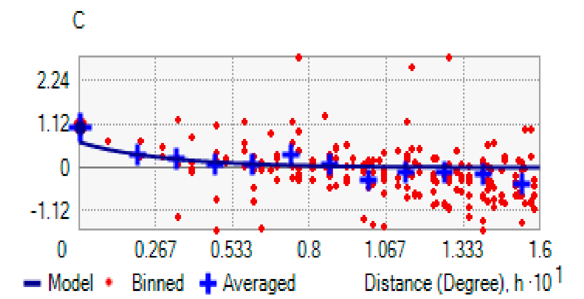

Cr

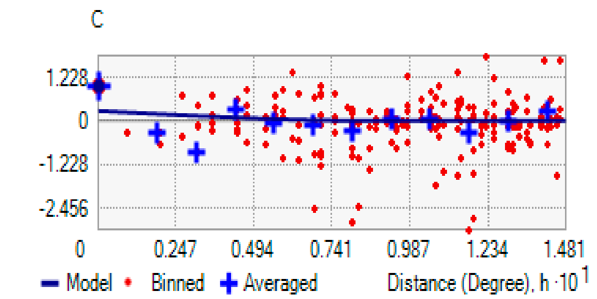

Cu

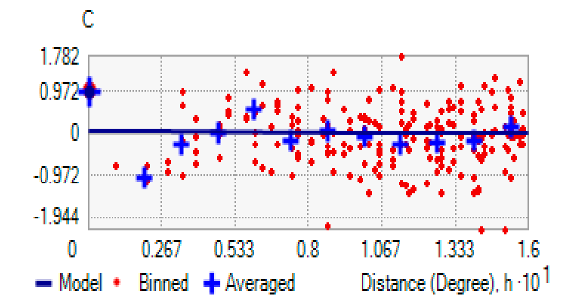

Mn

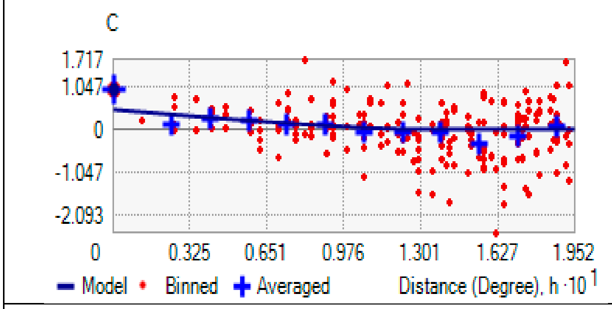

Ni

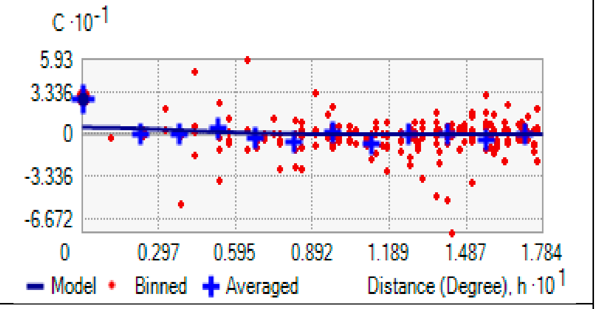

Pb

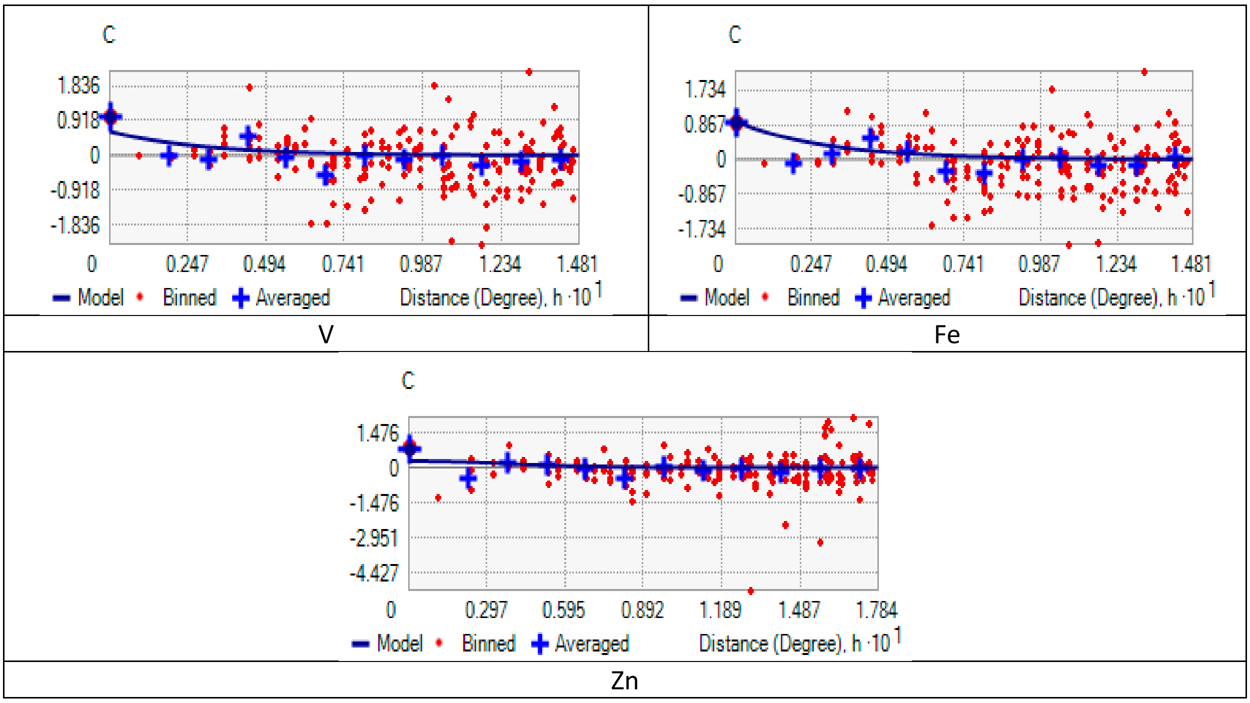

Figure S2. Semivariograms of studied elements.

Table S1. Limit of detection of studied elements.

elements	Concentrations	LOQ	LOD
As		0.1	0.03
Cd		0.1	0.03
Co		1	0.30
Cr		1	0.30
Cu		1	0.30
Mn	mg kg^{-1}	5	1.51
Ni		1	0.30
Pb		2	0.60
V		1	0.30
Fe		100	30.30
Zn		2	0.60

Table S2. Classification of contamination factor (CF).

**Contamination Factor Index (CF)**	< 1	Low contamination
	1 – 3	Moderate contamination
	3 – 6	Considerable contamination
	> 6	Very high contamination

Table S3. Classification of pollution load index (PLI).

PLI value	Classification
PLI < 1	Unpolluted
1 < PLI < 2	Moderately polluted
2 < PLI < 10	Strongly polluted
PLI > 10	Extremely polluted

Table S4. KMO and Bartlett's Test for studied variables.

KMO and Bartlett's Test

Kaiser-Meyer-Olkin Measure of Sampling Adequacy.		0.554
Bartlett's Test of Sphericity	Approx. Chi-Square	232.717
	df	55
	Sig.	0.000
